# Dynamic Kinesiotherapy: A Novel Technique That Promotes Appropriate Abdominal Wound Healing

**DOI:** 10.7759/cureus.41121

**Published:** 2023-06-28

**Authors:** Andrzej Ratajczak, Małgorzata Ratajczak-Lange, Kinga Zastawna, Abdalla Saad Abdalla Al-Zawi, Tomasz Banasiewicz

**Affiliations:** 1 General and Endocrinological and Gastroenterological Oncology Surgery, Poznan University of Medical Sciences, Poznań, POL; 2 General and Breast Surgery, Mid and South Essex University Hospital Group, Basildon, GBR; 3 General and Breast Surgery, Basildon and Thurrock University Hospital, Basildon, GBR; 4 General and Breast Surgery, Anglia Ruskin University, Chelmsford, GBR

**Keywords:** eventration, negative pressure wound therapy, laparatomy, ­wound healing, dynamic kinesiotherapy

## Abstract

Appropriate wound healing is a crucial phase of surgical patients’ management, as it facilitates early mobilization and return to everyday life. Also, it is required before offering patients the next phase of treatment. Surgical wound closure techniques have evolved remarkably; they include simple sutures, surgical clips, and adhesive compounds. Some appliances are used to support the closed surgical wounds and assist the abdominal wall muscles’ movements; however, they may make breathing uncomfortable or cause pain. Here, we introduce a novel, effective technique that facilitates appropriate surgical wound healing. Dynamic kinesiotherapy is an easy procedure to perform, handy, beneficial, safe, and cost-effective.

## Introduction

Abdominal wall closure after a laparotomy is an important aspect of surgery. Poor technique, inappropriate selection of suturing materials, as well as inexperience, can all increase the risk of serious postoperative complications. The evidence reflecting those issues was explored in 2015 by the European Hernia Society, and a Guidelines Development Group was created to provide recommendations on the closure of abdominal wall incisions. The recommendations support the utilization of techniques that reduce the rate of incisional hernias, such as not utilizing the midline approach to a laparotomy whenever possible, performing a continuous suturing technique, and to avoid the use of rapidly absorbable sutures. Also, there is a call for further work to research the impact of prescribing abdominal binders on the occurrence of incisional hernias [1].

In this research, we present an original scientific concept that supports standard surgical techniques by using dynamic kinesiotherapy. Our suggested method, a simple surgical taping technique, is not only prophylactic after laparotomies, but is especially useful in the treatment of wound dehiscence, any “open abdomen” procedures, as well as incisional hernias.

## Technical report

Our recommended method is a taping technique used to support the primary wound closure of various abdominal incisions after laparotomies. We used cotton-based, hypoallergenic, waterproof, air-permeable tape, certified by the Food and Drug Administration (FDA) and registered as a medical product with the CE marking (conformity with European health, safety, and environmental protection standards). The tape’s main advantage is its flexibility; it is adapted to truncal movements to facilitate easy mobilization. By counteracting the tensile forces generated by the contracting oblique and transverse abdominal muscles, it reduces the chances of lateralization (the main risk factor for wound dehiscence). We can think of this as using wound-closure strips (colloquially known as Steri-Strips) on a wound, only on a macro level.

After the complete laparotomy wound closure, and prior to taping, a dressing of the surgeon’s choice is applied to the wound. The use of taping with a vacuum dressing, such as closed incision negative pressure wound therapy (ciNPWT) in selectively infected wounds, is also possible and has been successfully applied in the author’s clinical practice. The tape is placed over the dressing. The skin should be clean and dry before tape application.

**Figure 1:**
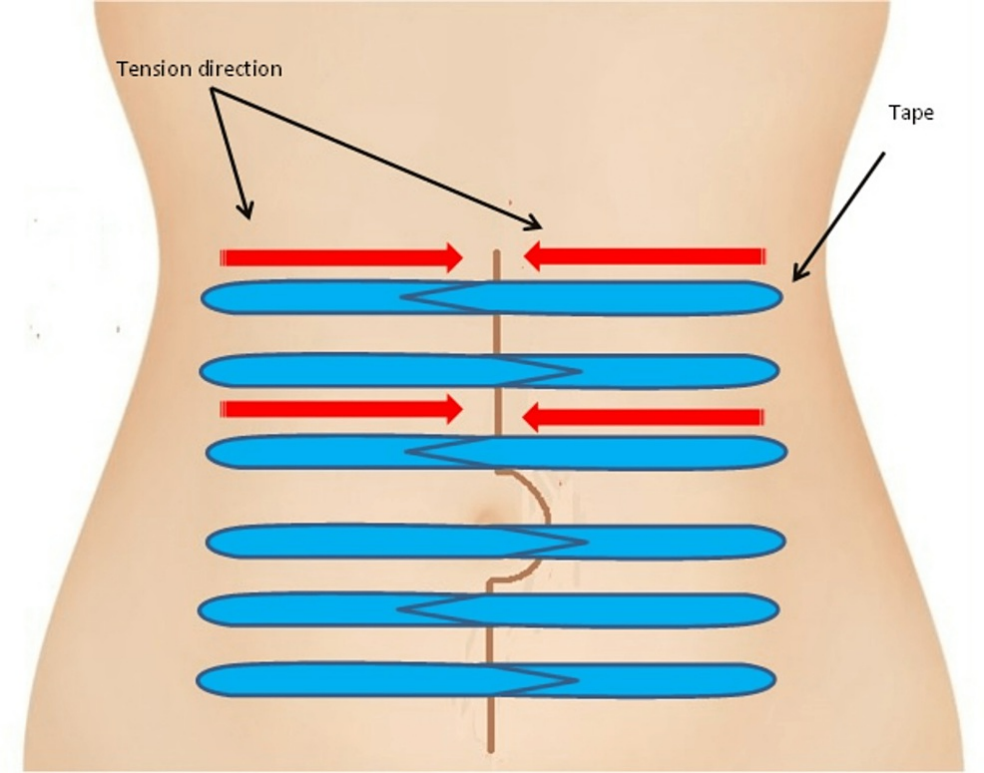
Dynamic kinesiotherapy, pair of tapes applied symmetrically and concurrently to both sides of the incision with lateral to medial traction. The illustration had been created by the author Dr. Abdalla Saad Abdalla Al-Zawi.

In case of vertical incisions (clean, clean-contaminated, or contaminated wounds), we start at the mid-axillary line; the tape is applied laterally to medially - with tension - towards the surgical incision, but not completely. The center portion of the tape must not be applied to the wound yet. Beforehand, another tape must be applied symmetrically to the opposite side of the incision. Only then can the center of the tape be glued together, resulting in an incision that is protected from the stretching and shearing abdominal muscle forces (Figure 1). This taping method is performed in the same manner along the entire incision. It is vital to begin taping at the very top of the surgical wound to relieve the tension of the whole surgical incision. The interval between the individual successive tape strips is up to the individual but ideally should not exceed 2 cm (approximately ¾ of an inch) in size.

**Figure 2:**
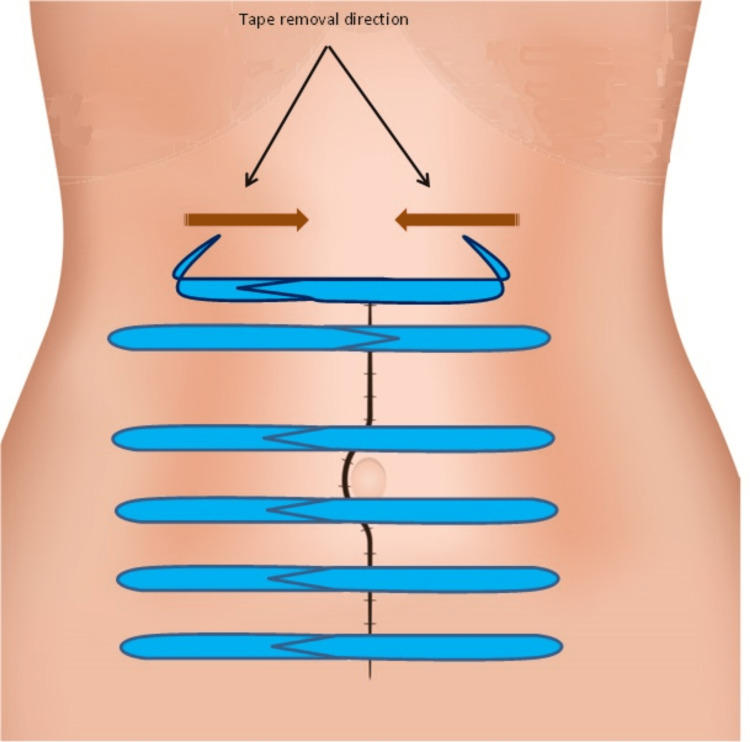
Tape removal by simultaneously detaching both outer ends of the tape strips and pulling towards the wound center at the same time. The illustration was created by the author Dr. Abdalla Saad Abdalla Al-Zawi.

In case of wound dressing change (due to contamination or dressing peeled off), the tapes have to be removed and replaced also. It is crucial to remove both opposing tape strips simultaneously. This is done by detaching both outer ends of the tape strips and pulling towards the center at the same time; thus, the lateral-medial stretching forces are completely omitted (Figure 2).

This taping technique is not limited to a midline laparotomy incision. With oblique or transverse abdominal incisions, it is important to apply the tape strips as per the instructions above, perpendicularly to the surgical incision.

This taping treatment should be avoided in patients with skin lesions on the operated area and in those with a known glue allergy. Prior to discharging the patient from the hospital, patients are educated on proper taping for 30 postoperative days. The tape can be used while bathing and showering.

## Discussion

One of the key elements required for proper surgical wound healing is the appropriate closure technique choice, regardless of how long the wound is or where it is located. The unique surgical anatomy of the anterior abdominal wall must be taken into consideration. Although the myofascial elements form a strong and durable structure, they have to remain flexible to allow a complete and effective range of movement, which includes truncal twisting and flexion. Also, together with the diaphragm, these muscles play a key role in breathing.

The antagonistic functions of paired muscles on either side of the anterior abdominal wall allow appropriate comprehensiveness of the functions above, but if the anterior abdominal muscles’ integrity is disrupted, serious consequences may arise. Unbalanced eccentric muscle contraction of the transverse and oblique muscles may cause the anterior abdominal wall compartments to spread sideways, leading to progressive lateralization of the rectus muscle and increasing fascial defects. This may lead to visceral prolapse through anterior abdominal wall defects, as is observed in large ventral hernias. This is especially true in “loss of domain” cases [2]. Such a situation may be aggravated by an unfavorable elevation in intra-abdominal pressure or obesity. It may result in other systemic complications such as shifting the body’s center of gravity with lumbar hyperlordosis, spinal degeneration, and exacerbation of coexisting chronic pain. Visceral displacement leads to ptosis of the diaphragm [3], which may interfere with breathing functions; thus, herniologists are fearful of respiratory failure as a major postoperative complication after large incisional hernia repair, this is why it is crucial to optimize primary wound closure after laparotomy. Finally, the elongated and stretched mesentery of the displaced intestine can cause inadequate blood supply; consequently, chronic ischemia of hernial sac contents is frequently observed. Patients are also at risk of chronic constipation due to the disrupted passage of intestinal contents. Additionally, there is a risk of incarceration of a hernia, leading to vessel strangulation and bowel necrosis. Trophic changes and ulcerations, which often become infected, are frequent in particular when skin over a large hernia has been stretched for a long period.

Apart from hernias, which always develop as a late consequence of postoperative wound complications, another serious complication occurring in the immediate postoperative period is eventration, where the fascia separates and herniated bowels are protruded through the postoperative wound, either under the skin or totally outside the body. This exposes the patient to subsequent surgery, prolonged hospitalization, and delayed recovery. Each subsequent closure of the abdominal wall is increasingly difficult and carries risks of further complications.

During the postoperative period, clinicians may face challenges such as cough, ascites, hypoalbuminemia, a genetic predisposition to collagen defects, or collagen destruction due to nicotinism [4,5]. Other conditions that need to be taken into consideration include diabetes, jaundice, steroid therapy, obesity, and cancer.

Finally, the most feared complication in patients after numerous open abdominal interventions is the “frozen abdomen”. Wound dehiscence can lead to the development of infection, inflammation, and adhesions between the anterolateral abdominal wall and abdominal viscera; this is often complicated further with intestinal fistulas [6]. These patients require non-standard approaches to treatment, including negative pressure, biological patches [7], or separation techniques such as transversus abdominis release (TAR) and abdominal decompression in abdominal compartment syndrome (ACS). A commonly used method in this group of patients is negative pressure therapy. It has been shown that during its application, it is of utmost urgency for primary fascial closure to occur as soon as possible. Another closure technique is called the abdominal re-approximation anchor device (ABRA) technique. This technique is based on retention sutures that are placed outside of the primary suture line through all layers of the abdominal wall, including the skin. The sutures gradually approximate the retracted skin defects through chronic cyclic tension to facilitate primary wound closure [8]. Taping, which can assist in the approximation of the wound edges without limiting fascial mobility as long as there is no "frozen abdomen", is a logical complement to these strategies.

No significant side effects were observed with this taping technique. The only, fortunately minor, complication observed was an allergy to the tape glue. This sensitivity can be avoided with the use of high-quality hypoallergenic tape, which is only slightly more expensive than the standard version. The cost of one roll of tape - sufficient to completely supply a patient for the duration of healing/treatment - is extremely cost-effective, as it does not exceed 10 EUR (~11 USD). Unlike the widely used postoperative belts, the tape does not compress the entire abdomen, but rather exerts selective traction on the abdominal integument, pulling it medially, relieving the postoperative wound of unnecessary pressures, avoiding peritoneal cavity compression, and causing no impact on breathing or mobility. When assessing patients with the visual analogue scale (VAS), a pain rating scale, we noticed that coughing is not associated with as much pain as in other post-laparotomy patients. The ability to cough deeply allows optimal ventilation of the lower lungs and effective expectoration of secretions, thus preventing atelectasis and postoperative pneumonia. By assessing postoperative pain on the VAS scale, which has a subjective pain bias for each patient, we conclude that this taping technique causes a reduction in postoperative pain. Another significant lifestyle advantage of tape is that it is waterproof, so patients can freely bathe and shower. Additionally, there are no movement limitations and therefore no risk of abdominal wall muscular atrophy, as exists with prolonged use of a hernia belt. The psychological effect of the taping technique cannot be underestimated, as patients in the postoperative period feel safe and are willingly able to self-care after hospital discharge.

## Conclusions

In summary, there are only advantages to using this taping technique: simplicity, ease of use in all laparotomy situations, and cost-effectiveness, as well as no contraindications, patient limitations, side effects, or complications. The application of tape takes a mere 1-2 minutes and can be accomplished by a nurse or by patients themselves after hospital discharge. We believe that the taping technique described here fits ideally with the basic principle that should guide every doctor - primum non nocere (first, do no harm). We are hopeful that our idea will revolutionize, and simplify, the approach to postoperative wound treatment. Of course, we are aware there is a lack of prospective, randomized clinical evaluations confirming our assumptions about the decrease in postoperative complications (including the number of hernias, dehiscence, pneumonia, etc.). Such assessments and full evaluations will take some time; however, we are eager to share our preliminary findings, which we strongly believe will benefit many patients.

We have found no literature on this topic in available databases. The few reports on kinesiotherapy, written by physiotherapists, describe a completely different use of tape.
